# Postmortem CT Angiography Compared with Autopsy: A Forensic Multicenter Study

**DOI:** 10.1148/radiol.2018170559

**Published:** 2018-05-01

**Authors:** Silke Grabherr, Axel Heinemann, Hermann Vogel, Guy Rutty, Bruno Morgan, Krzysztof Woźniak, Fabrice Dedouit, Florian Fischer, Stefanie Lochner, Holger Wittig, Giuseppe Guglielmi, Franziska Eplinius, Katarzyna Michaud, Cristian Palmiere, Christine Chevallier, Patrice Mangin, Jochen M. Grimm

**Affiliations:** From the University Center of Legal Medicine Lausanne-Geneva, Chemin de la Vulliette 4, 1000 Lausanne 25, Switzerland (S.G., F.D., K.M., C.P., C.C., P.M., J.M.G.); Institute of Legal Medicine, University Medical Center Hamburg-Eppendorf, Hamburg, Germany (A.H., H.V.); East Midlands Forensic Pathology Unit, University of Leicester, Leicester, England (G.R.); University of Leicester Imaging Department, University Hospitals of Leicester, Leicester Royal Infirmary, Leicester, England (B.M.); Department of Forensic Medicine, Jagiellonian University Medical College, Krakow, Poland (K.W.); Departments of Legal Medicine and Radiology, Hôpital Rangueil, Toulouse, France (F.D.); Institute of Legal Medicine, Munich, Germany (F.F., S.L.); Institute of Legal Medicine, Basel, Switzerland (H.W.); University of Foggia, Foggia, Italy (G.G.); Institute of Forensic Medicine, University of Leipzig, Leipzig, Germany (F.E.); and Department of Medical Radiology, University Hospital Center and University of Lausanne, Lausanne, Switzerland (J.M.G.).

## Abstract

**Purpose:**

To determine if postmortem computed tomography (CT) and postmortem CT angiography help to detect more lesions than autopsy in postmortem examinations, to evaluate the strengths and weaknesses of each method, and to define their indications.

**Materials and Methods:**

Postmortem CT angiography was performed on 500 human corpses and followed by conventional autopsy. Nine centers were involved. All CT images were read by an experienced team including one forensic pathologist and one radiologist, blinded to the autopsy results. All findings were recorded for each method and categorized by anatomic structure (bone, organ parenchyma, soft tissue, and vascular) and relative importance in the forensic case (essential, useful, and unimportant).

**Results:**

Among 18 654 findings, autopsies helped to identify 61.3% (11 433 of 18 654), postmortem CT helped to identify 76.0% (14 179 of 18 654), and postmortem CT angiography helped to identify 89.9% (16 780 of 18 654; *P* < .001). Postmortem CT angiography was superior to autopsy, especially at helping to identify essential skeletal lesions (96.1% [625 of 650] vs 65.4% [425 of 650], respectively; *P* < .001) and vascular lesions (93.5% [938 of 1003] vs 65.3% [655 of 1003], respectively; *P* < .001). Among the forensically essential findings, 23.4% (1029 of 4393) were not detected at autopsy, while only 9.7% (428 of 4393) were missed at postmortem CT angiography (*P* < .001). The best results were obtained when postmortem CT angiography was combined with autopsy.

**Conclusion:**

Postmortem CT and postmortem CT angiography and autopsy each detect important lesions not detected by the other method. More lesions were identified by combining postmortem CT angiography and autopsy, which may increase the quality of postmortem diagnosis.

*Online supplemental material is available for this article.*

## Introduction

With the introduction of modern cross-sectional imaging techniques such as multi–detector row computed tomography (CT) and magnetic resonance (MR) imaging to postmortem investigations, forensic pathology has taken an important step forward (1,2). Relatively low maintenance costs, short examination times, and ease of operation make CT a widely used cross-sectional imaging technique in modern postmortem imaging (3). Compared with conventional autopsy, postmortem CT has several advantages, which can lead to important improvements in both research and postmortem investigation (4–8). The main reported weakness of postmortem CT, however, is relatively low soft-tissue contrast, especially in organ parenchyma, and poor ability to view the vascular system (8). Cardiovascular disease is a major cause of unexpected natural death in most developed countries (9), and this limitation decreases the potential of postmortem CT to help diagnose cardiovascular disease. Consequently, the reference standard for investigation of natural death, and particularly cardiovascular death, is considered to be conventional autopsy (2–5,7).

In clinical radiology, these limitations are addressed with the use of intravenous contrast agents. Consequently, various postmortem angiographic techniques have been developed (10–19). Probably the most widespread single approach for postmortem angiography today is multiphase postmortem CT angiography (20–27), first described in 2011 (14). This technique uses a standardized procedure on the basis of a defined injection and a scanning protocol that uses a specific perfusion device and an oil-based contrast agent of specific viscosity. A previous study (8) revealed that the addition of postmortem CT angiography to postmortem CT increased the sensitivity for detecting pathologic findings from 64% to approximately 81%, which is comparable to the sensitivity of conventional autopsy (∼83%). Because this study was conducted in only one center of forensic medicine and thus it involved a small number of human corpses, the technical working group for postmortem angiography methods decided in 2012 to initiate a multicenter study with the goal of validating the technique of multiphase postmortem CT angiography on a large number of cases.

The purpose of this study was to determine if postmortem CT and postmortem CT angiography help to detect more lesions than autopsy in postmortem examinations, to evaluate the strengths and weaknesses of each method, and to define their indications.

## Materials and Methods

### Study Design

The study was a prospective multicenter study. Nine European centers participated and acquired data from unenhanced postmortem CT, postmortem CT angiography, and conventional autopsy on 500 human corpses in which an autopsy was ordered. The study was conducted from February 2012 to August 2015. Inclusion criteria were as follows: subject age older than 16 years, the performance of all three examinations in accordance with the standardized protocol for every case, and a fully recorded data set.

This study was partially funded by a private company (Fumedica AG, Muri, Switzerland). The funding included travel expenses, cost of tubing sets and contrast agent, rental for a Virtangio device for the duration of the study (for centers in Foggia, Italy; Krakow, Poland; Leipzig and Munich, Germany; Toulouse, France; Leicester, England; and Basel, Switzerland).

### Study Preparation

Where required, the approval of local ethics committees was obtained (depending on the country or previously existing agreements) in accordance with local legislation. The examinations from England additionally received consent from the next of kin. Training in postmortem CT angiography was provided in a preparation phase by the principal investigator’s center to each attending center, including at least 1 week of training in the performance of postmortem CT angiography.

### Data Acquisition

CT images were acquired according to standardized scanning protocols adapted to the equipment at each center. An overview of the CT parameters is as follows: section thickness, 0.75–3 mm; spacing interval, 0.6–2 mm; field of view, 414–500 mm; tub voltage, 110–130 mm; tube current, 100–380 mA; and standard algorithm of reconstruction. Details are provided in Table E1 (online). Postmortem CT angiography was performed by using the standardized protocol described by Grabherr et al (14). This included the application of specific single-use sets for femoral vascular cannulation and the injection of a contrast agent mixture of an oil-based solution of defined viscosity (mixture of paraffin oil [paraffinum liquidum] with 6% oil-based contrast agent [Angiofil; Fumedica, Muri, Switzerland]) by using a special perfusion device (Virtangio; Fumedica, Muri, Switzerland) and the standardized injection protocol, including an arterial, venous, and dynamic phase of injection.

Conventional forensic autopsy was performed on each body by the forensic pathologists in charge of the case in accordance with local and European requirements and standards (examination of the cranial, thoracic, and abdominal cavities) (28). These experts were informed of the most important radiologic findings before the autopsy was performed, which enabled them to adequately adapt their autopsy technique except in England, where autopsy was performed according to Royal College of Pathologists guidelines (29) independent of the radiologic results. A complete autopsy report was provided by the lead forensic pathologists.

### Data Registration and Analysis

A team from the coordinating study center evaluated the data from the included cases (S.G., a board-certified forensic pathologist with 10 years of experience interpreting radiologic data, particularly postmortem CT angiography data; J.M.G., a board-certified radiologist with 5 years of experience reading postmortem CT and postmortem CT angiography data; and P.M., K.M., and C.P., all board-certified forensic pathologists with 15–30 years of experience in the field and fluent in the local language of the visited center). The first two team members read the radiologic images of the included bodies (postmortem CT and postmortem CT angiography) and entered every pathologic finding into a spreadsheet. The second forensic pathologist extracted all pathologic findings reported by the local forensic pathologists from the autopsy report. All findings described at postmortem CT, postmortem CT angiography, and autopsy were then entered in a spreadsheet (Excel; Microsoft, Redmond, Wash) together with background case information (ie, age, sex, and circumstances of death).

Regarding the circumstances of death, we grouped cases into four groups on the basis of the cause of death and the indication of the postmortem examination: natural death (eg, cardiovascular incident and sepsis); polytrauma (severe multisystem injury such as fall from great height or traffic accident); other violent death (localized injuries such as ballistic trauma, sharp force trauma, and blunt force trauma); and suspected medical error (death during or shortly after a medical intervention in which a postmortem examination was performed to prove or rule out a medical error).

The different findings were jointly classified by two board-certified forensic pathologists from the coordinating study center (S.G. and either K.M., P.M., or C.P.) into three groups (essential, useful, or unimportant) on the basis of their importance to the forensic investigation of the case, such as their contribution toward identifying the cause of death, the events that led up to the death, and reconstruction of the forensic background. For example, in a cardiac death, coronary plaques, stenoses, and the associated myocardial infarction would be considered essential; other vascular pathologic analyses would be considered useful; and an old appendectomy or hip prosthesis would be considered unimportant. Findings were also categorized anatomically as bone, soft tissue (including muscles, tendons, connective and fatty tissue, and the skin), parenchyma (parenchymatous organs, intestines, and the heart), and vascular (calcification, stenosis, occlusion, aneurysm, and rupture of vessels including the coronary arteries).

Findings of additional examinations and analyses before or after imaging and/or autopsy (most importantly external examination, histologic analysis, and toxicology analysis) were not considered in this study because they can be performed independently or combined with both conventional autopsy and modern imaging methods.

### Statistical Analysis

Statistical analyses were performed by using statistical software (MedCalc version 15.6.1, MedCalc Software, Ostend, Belgium; and SPSS version 25, IBM, Armonk, NY). Postmortem CT, postmortem CT angiography, and autopsy results were compared by using Cochran *Q* test. The required difference between groups for pairwise comparisons was determined according to Sheskin if Cochran *Q* test showed a statistically significant result (30). Values presented in the text were additionally evaluated with McNemar test by using the simple sampling bias corrected accelerated bootstrap method with 1000 samples. A *P* value of less than .05 was considered to indicate statistical significance.

## Results

Autopsy was performed on the day of the postmortem CT and postmortem CT angiography examination, or the following day, in all cases. The maximum interval between death and autopsy was 5 days. After examination of 500 bodies, a total of 18 654 findings were recorded. Demographic data are in Table 1. Results of the comparisons between postmortem CT, postmortem CT angiography, and autopsy are in Table 2. Further details regarding the stratification of results across sites and for different manners of death can be found in Tables E2 and E3 (online). Diagrams that display the cumulative advantage of autopsy, postmortem CT, and postmortem CT angiography are in Figure 1.

**Table 1:**
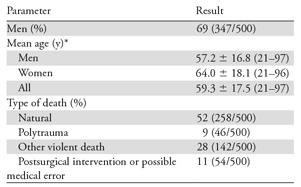
Study Demographics

Note.—Unless otherwise indicated, data in parentheses are numerator/denominator.

*Data are ± standard deviation; data in parentheses are range.

**Table 2:**
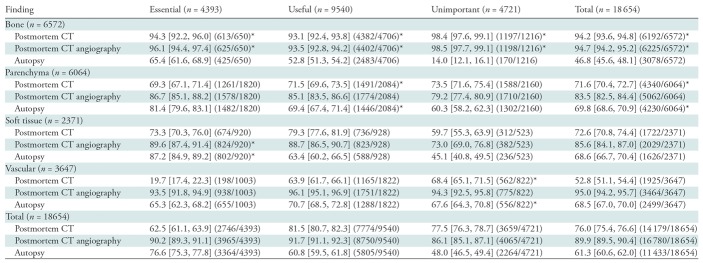
Findings Detected according to Method

Note.—Data are percentages; data in brackets are 95% confidence intervals; and data in parentheses are numerator/denominator. Unless otherwise indicated, each method had a difference that was significant versus the other two methods. *P* values per Cochran *Q* tests were less than .001. Parenchyma refers to parenchymatous organs, intestines, and the heart. Soft tissue refers to muscles, tendons, connective and fatty tissue, and the skin.

*Statistical significance (*P* < .05) of the larger of the differences from the respective two other methods found by using pairwise comparison.

**Figure 1a:**
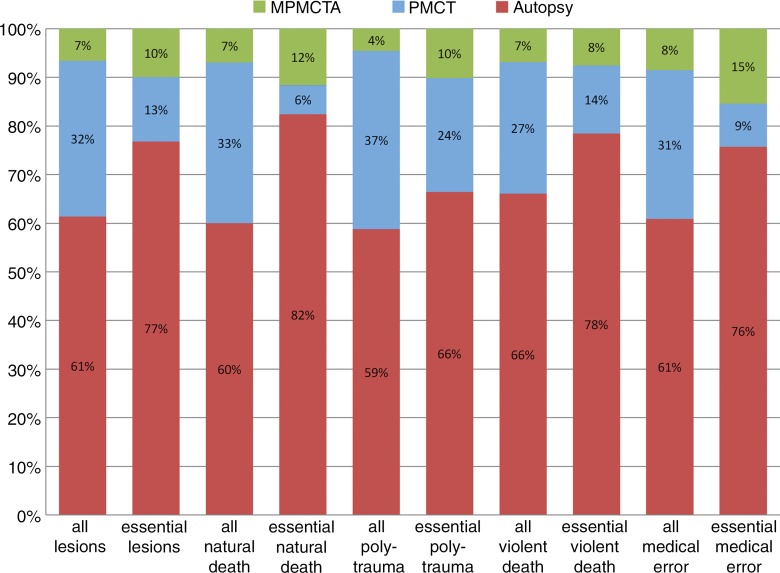
Graph of additional postmortem findings. **(a)** Additional findings obtained by using imaging with autopsy. Autopsy findings as a percentage of all findings are in red. Additional findings not observed at autopsy but identified at postmortem CT are in blue. Green indicates findings undiscovered at both autopsy and postmortem CT but detected at postmortem CT angiography. There is a relatively high effect of postmortem CT in polytrauma evaluation and of postmortem CT angiography in medical errors and natural death. **(b)** Additional findings obtained by using angiography with postmortem CT, and then by performing an autopsy. Postmortem CT findings as a percentage of all findings are in blue. Additional findings detected by using postmortem CT angiography are in green. Finally, findings only detected at autopsy are in red. Notice the relatively high effect of autopsy in medical errors and natural death, and the low effect in evaluation of polytrauma.

**Figure 1b:**
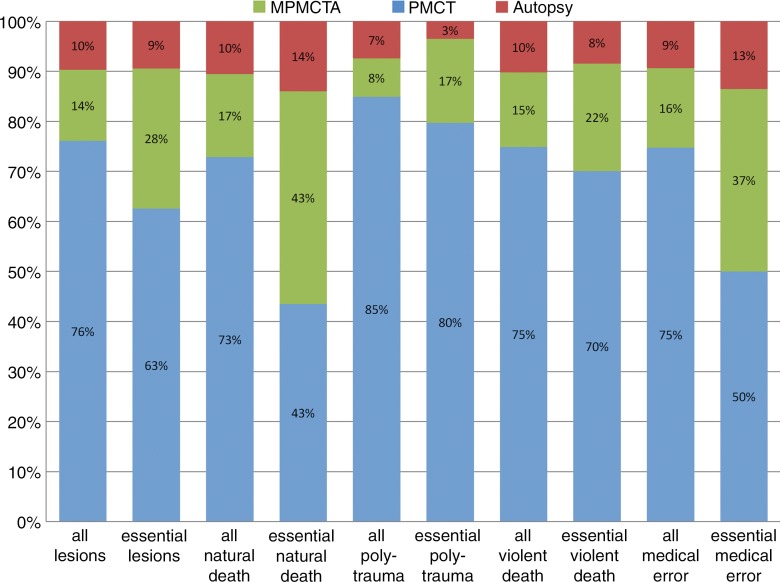
Graph of additional postmortem findings. **(a)** Additional findings obtained by using imaging with autopsy. Autopsy findings as a percentage of all findings are in red. Additional findings not observed at autopsy but identified at postmortem CT are in blue. Green indicates findings undiscovered at both autopsy and postmortem CT but detected at postmortem CT angiography. There is a relatively high effect of postmortem CT in polytrauma evaluation and of postmortem CT angiography in medical errors and natural death. **(b)** Additional findings obtained by using angiography with postmortem CT, and then by performing an autopsy. Postmortem CT findings as a percentage of all findings are in blue. Additional findings detected by using postmortem CT angiography are in green. Finally, findings only detected at autopsy are in red. Notice the relatively high effect of autopsy in medical errors and natural death, and the low effect in evaluation of polytrauma.

When viewed in conjunction, the diagrams show that about a quarter to a fifth of the essential and useful findings is missed at autopsy. When postmortem CT is performed in addition to autopsy, the proportion of missed findings is reduced to about 10%. Roughly the same amount and quality of information is delivered by combining postmortem CT and postmortem CT angiography. The best results are achieved when autopsy is combined with postmortem CT and postmortem CT angiography, especially in cases of natural death and malpractice.

The greatest advantage of postmortem CT or postmortem CT angiography over autopsy was observed for the detection of bone and vascular lesions for any manner of death (Table 2). For both essential findings and all findings, only 9.7% (428 of 4393) and 10.0% (1874 of 18 654), respectively (*P* < .001), would have been overlooked if postmortem CT angiography had been performed without autopsy. If autopsy had been performed without postmortem CT or postmortem CT angiography, 38.7% of all findings (7221 of 18 654) and 23.4% of essential findings (1029 of 4393) would not have been reported (*P* < .001). If only postmortem CT had been performed, 24.0% of all findings (4475 of 18 654) and 37.5% of essential findings (1647 of 4393) would have remained unreported (*P* < .001).

In cases of natural death (Table E3a [online]), only about half of the essential bone lesions (53.8%; seven of 13) and two-thirds of essential soft tissue lesions (64.0%; 32 of 50) were detected at autopsy, whereas postmortem CT angiography helped to detect 84.6% (11 of 13; *P* > .05) and 90.0% (45 of 50; *P* < .05), respectively. This superiority was less pronounced for essential vascular lesions (autopsy vs postmortem CT angiography, 79.3% [472 of 595] vs 90.8% [540 of 595], respectively; *P* < .001) whereas more essential parenchyma lesions were detected at autopsy (autopsy vs postmortem CT angiography, 85.9% [593 of 690] vs 81.0% [559 of 690], respectively; *P* < .05).

In cases of violent death (Table E3b [online]), more than half of the essential vascular findings were missed at autopsy (60.9%; 95 of 156). At postmortem CT, 87.8% (137 of 156) of essential vascular findings were missed, whereas less than 1% (one of 156) were missed at postmortem CT angiography (all *P* < .001). However, autopsy showed a slightly higher detection rate for essential soft-tissue lesions versus postmortem CT angiography (90.2% [642 of 712] vs 88.6% [631 of 712], respectively), which was not statistically significant.

In cases of polytrauma (Table E3c [online]), more than two-thirds of essential vascular lesions were not detected at autopsy (68.6%; 81 of 118). Only 5.9% of these (seven of 118) were detected at postmortem CT, whereas all were detected at postmortem CT angiography (all *P* < .001). Both postmortem CT and postmortem CT angiography performed better for the detection of essential bone and parenchyma findings (*P* < .005); however, for essential soft-tissue lesions, postmortem CT angiography performed better than autopsy (*P* < .01). The difference with postmortem CT was not statistically significant.

In cases of suspected medical error (Table E3d [online]), 93.3% (125 of 134) of essential vascular lesions were detected at postmortem CT angiography. However, slightly less than a third (30.6%; 41 of 134) and two-thirds (63.4%; 85 of 134) of essential vascular lesions were detected at postmortem CT and autopsy, respectively (all *P* < .001).

## Discussion

Our results show that postmortem CT angiography is superior to autopsy for all findings except essential soft-tissue findings. The superiority of imaging over autopsy for the total and essential findings is strongly influenced by the significantly higher detection rates for bone and vascular lesions that together make up more than half of the findings. An example of a bone lesion, clearly visible at CT but not identifiable in autopsy, is shown in Figure 2. Whereas the bone lesions are already visible on the CT image, the vascular findings are rendered visible at CT angiography. Many of those vascular findings were not even visible in conventional autopsy, especially if small vessels were concerned. The advantage of postmortem CT angiography over autopsy and postmortem CT is therefore most notable when vascular pathologic changes are prevalent.

**Figure 2a:**
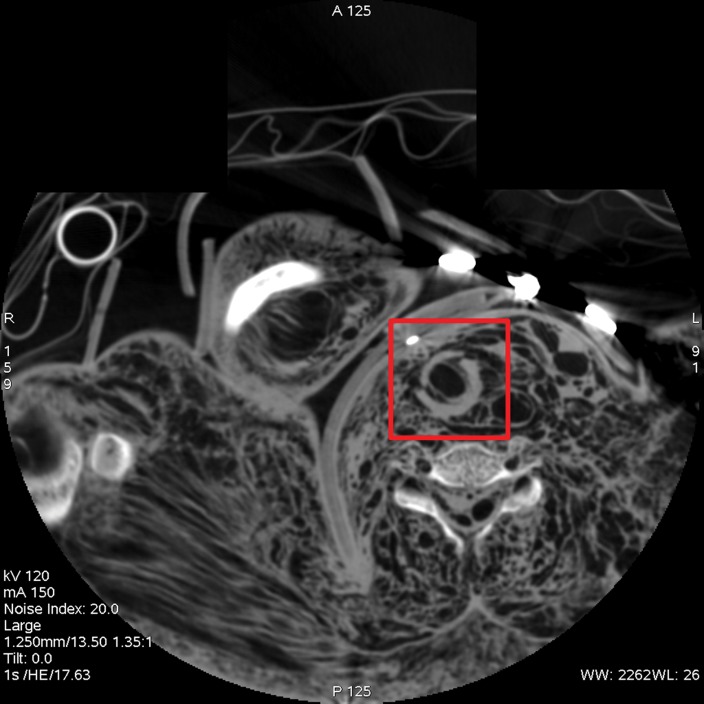
**(a)** Axial cervical postmortem CT scan and **(b)** zoomed section of the cricoid cartilage (box in **a**) of a 27-year-old woman who died of strangulation. Three-dimensional volume-rendered reconstructions from cranial (**c**), left lateral oblique (**d**), and right lateral oblique (**e**) views. Postmortem CT scan learly displays a displaced bilateral fracture of the cricoid cartilage (arrows). This finding is important because it proves the application of relevant force to the neck. It was difficult to demonstrate this finding at autopsy because anatomic preparation required extensive manipulation of the laryngotracheal region, which without postmortem CT would have been unclear regarding whether the fracture was caused by the preparation or was there before autopsy.

**Figure 2b:**
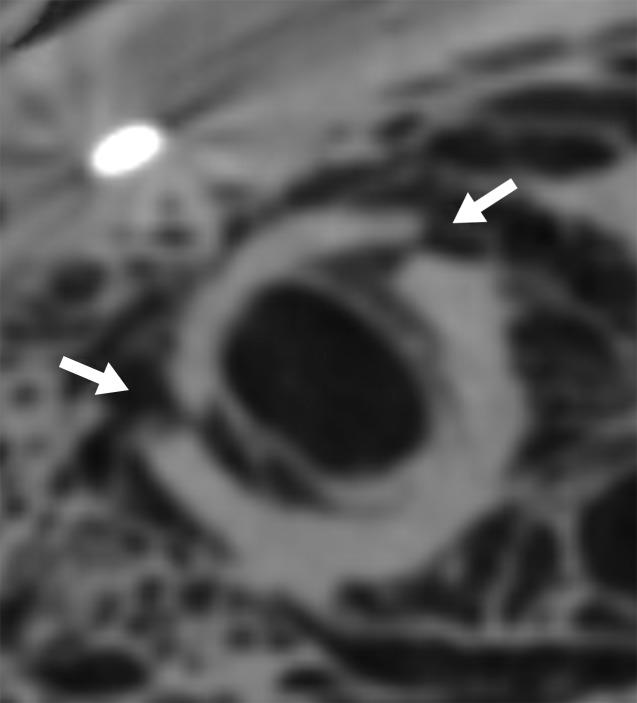
**(a)** Axial cervical postmortem CT scan and **(b)** zoomed section of the cricoid cartilage (box in **a**) of a 27-year-old woman who died of strangulation. Three-dimensional volume-rendered reconstructions from cranial (**c**), left lateral oblique (**d**), and right lateral oblique (**e**) views. Postmortem CT scan learly displays a displaced bilateral fracture of the cricoid cartilage (arrows). This finding is important because it proves the application of relevant force to the neck. It was difficult to demonstrate this finding at autopsy because anatomic preparation required extensive manipulation of the laryngotracheal region, which without postmortem CT would have been unclear regarding whether the fracture was caused by the preparation or was there before autopsy.

**Figure 2c:**
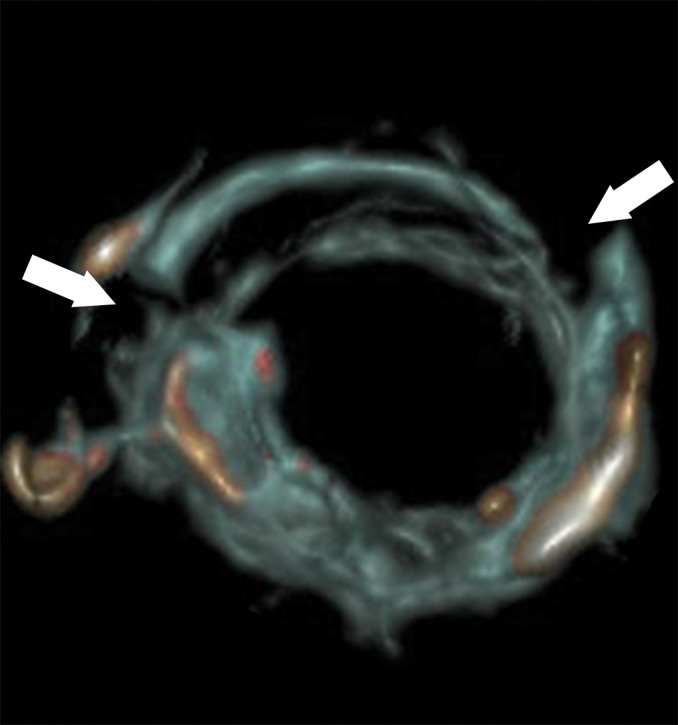
**(a)** Axial cervical postmortem CT scan and **(b)** zoomed section of the cricoid cartilage (box in **a**) of a 27-year-old woman who died of strangulation. Three-dimensional volume-rendered reconstructions from cranial (**c**), left lateral oblique (**d**), and right lateral oblique (**e**) views. Postmortem CT scan learly displays a displaced bilateral fracture of the cricoid cartilage (arrows). This finding is important because it proves the application of relevant force to the neck. It was difficult to demonstrate this finding at autopsy because anatomic preparation required extensive manipulation of the laryngotracheal region, which without postmortem CT would have been unclear regarding whether the fracture was caused by the preparation or was there before autopsy.

**Figure 2d:**
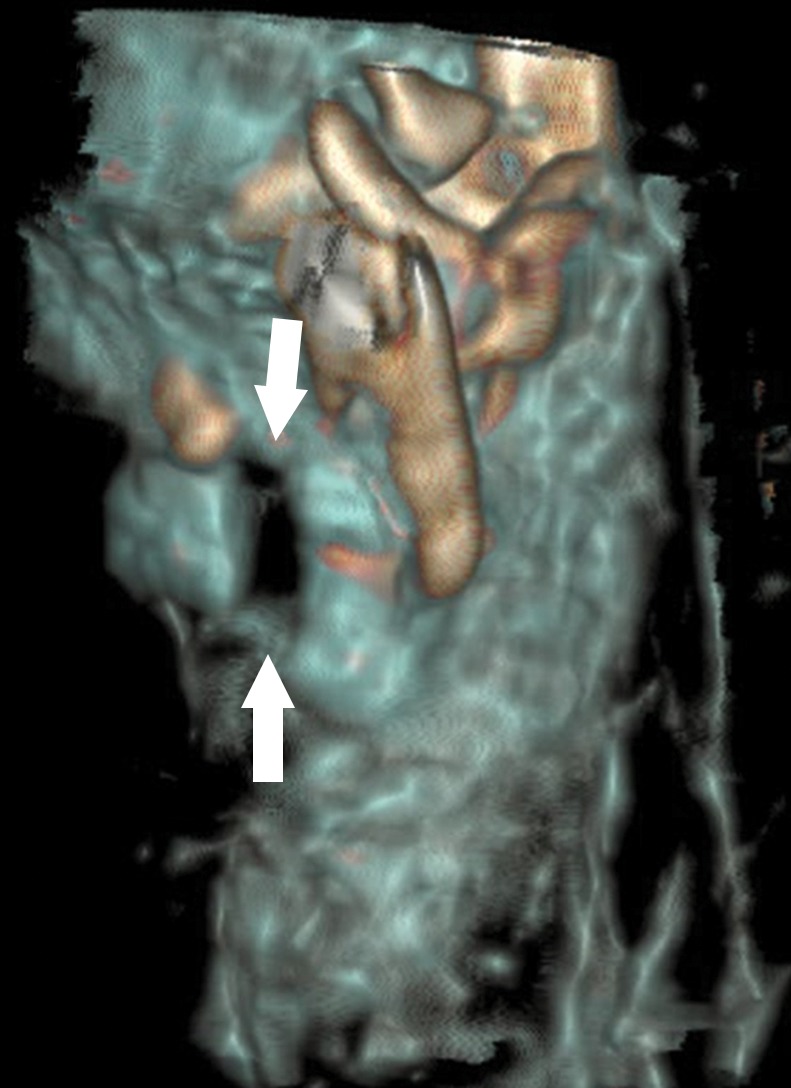
**(a)** Axial cervical postmortem CT scan and **(b)** zoomed section of the cricoid cartilage (box in **a**) of a 27-year-old woman who died of strangulation. Three-dimensional volume-rendered reconstructions from cranial (**c**), left lateral oblique (**d**), and right lateral oblique (**e**) views. Postmortem CT scan learly displays a displaced bilateral fracture of the cricoid cartilage (arrows). This finding is important because it proves the application of relevant force to the neck. It was difficult to demonstrate this finding at autopsy because anatomic preparation required extensive manipulation of the laryngotracheal region, which without postmortem CT would have been unclear regarding whether the fracture was caused by the preparation or was there before autopsy.

**Figure 2e:**
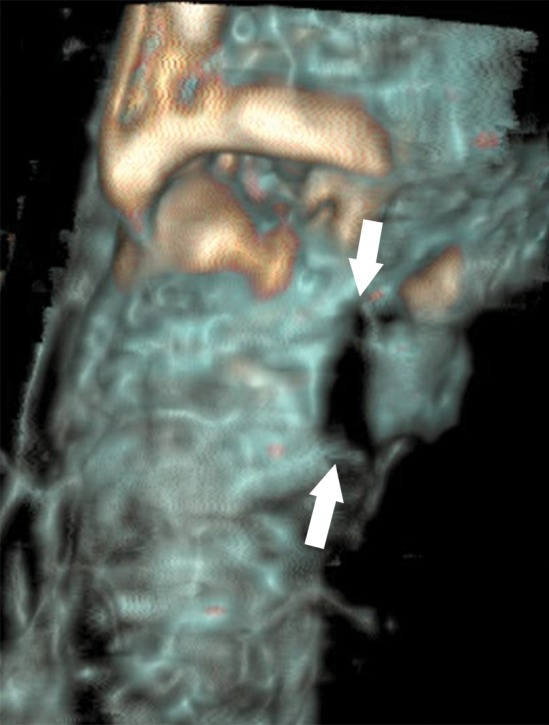
**(a)** Axial cervical postmortem CT scan and **(b)** zoomed section of the cricoid cartilage (box in **a**) of a 27-year-old woman who died of strangulation. Three-dimensional volume-rendered reconstructions from cranial (**c**), left lateral oblique (**d**), and right lateral oblique (**e**) views. Postmortem CT scan learly displays a displaced bilateral fracture of the cricoid cartilage (arrows). This finding is important because it proves the application of relevant force to the neck. It was difficult to demonstrate this finding at autopsy because anatomic preparation required extensive manipulation of the laryngotracheal region, which without postmortem CT would have been unclear regarding whether the fracture was caused by the preparation or was there before autopsy.

As expected, postmortem CT angiography resulted in additional findings compared with postmortem CT. The greatest number of additional essential findings were registered in cases of natural death and medical errors (Fig 3) and the majority of findings were categorized as vascular and parenchyma, for which postmortem CT angiography is especially sensitive. The cases in which postmortem CT angiography showed bone lesions that were not detected at postmortem CT (eight examinations) can be explained by bone contusions or compression fractures with visible contrast agent extravasation but no morphologic fracture signs.

**Figure 3a:**
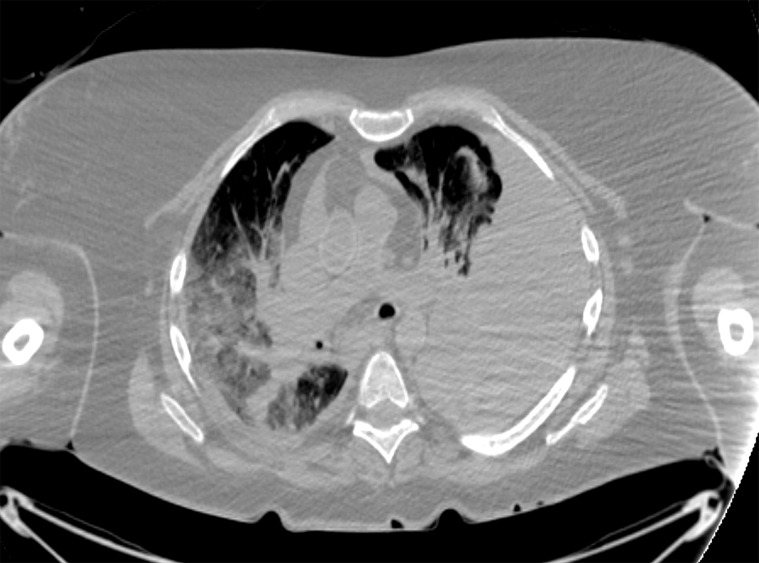
**(a)** Postmortem CT and **(b–d)** and arterial phase postmortem CT angiography images in a 59-year-old woman who died of internal exsanguination shortly after Whipple surgery. **(a)** A large left-sided hemothorax with mediastinal shift to the right. During the surgery, supraceliac clamping of the abdominal aorta was performed to stop intraperitoneal bleeding. The clamp was later loosened but left in place (arrow in **b**). The fatal hemothorax was caused by hemorrhage from the left 11th intercostal artery, which was torn near its origin from the aorta just above the diaphragm during placement of the clamp. The contrast media extravasation from the artery’s origin, reaching cranially into the thorax (arrows in **b**, **c**, and **d**), is displayed on arterial phase postmortem CT angiographic images (**c** and **d**). This finding may have been difficult to detect at autopsy because of the small size and location of the vessel. In this case it could not be displayed at autopsy because of large amounts of intraperitoneal and intrathoracic clotted blood, and multiple previous abdominal operations with extensive scar tissue formation and adhesions.

**Figure 3b:**
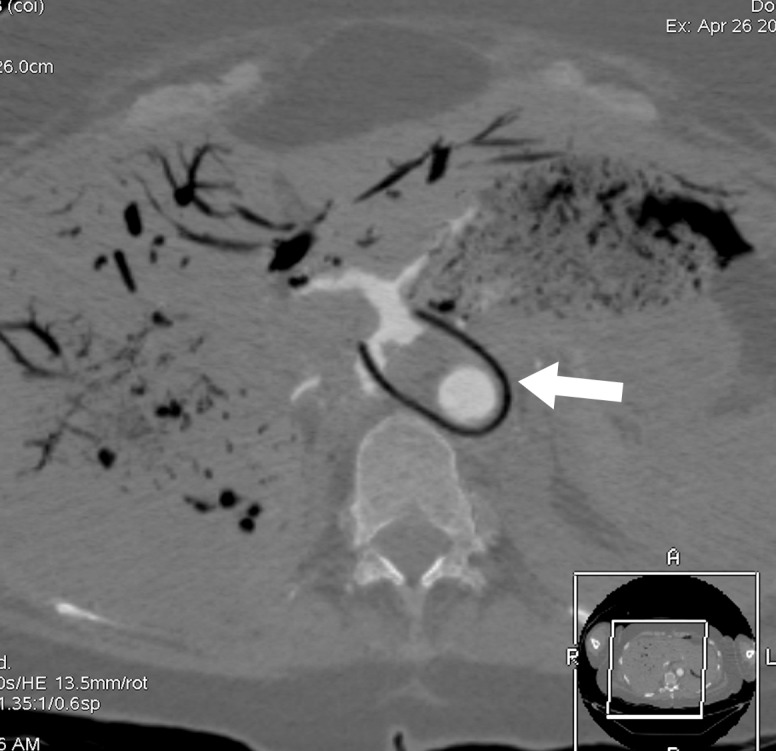
**(a)** Postmortem CT and **(b–d)** and arterial phase postmortem CT angiography images in a 59-year-old woman who died of internal exsanguination shortly after Whipple surgery. **(a)** A large left-sided hemothorax with mediastinal shift to the right. During the surgery, supraceliac clamping of the abdominal aorta was performed to stop intraperitoneal bleeding. The clamp was later loosened but left in place (arrow in **b**). The fatal hemothorax was caused by hemorrhage from the left 11th intercostal artery, which was torn near its origin from the aorta just above the diaphragm during placement of the clamp. The contrast media extravasation from the artery’s origin, reaching cranially into the thorax (arrows in **b**, **c**, and **d**), is displayed on arterial phase postmortem CT angiographic images (**c** and **d**). This finding may have been difficult to detect at autopsy because of the small size and location of the vessel. In this case it could not be displayed at autopsy because of large amounts of intraperitoneal and intrathoracic clotted blood, and multiple previous abdominal operations with extensive scar tissue formation and adhesions.

**Figure 3c:**
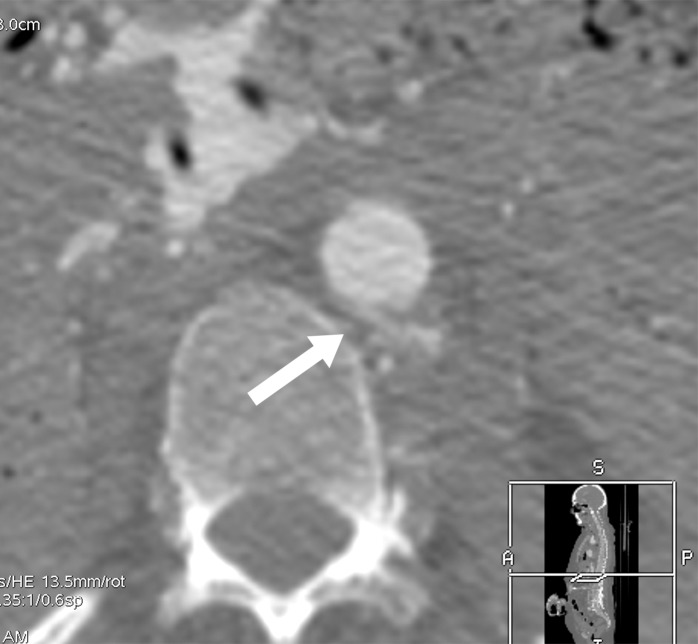
**(a)** Postmortem CT and **(b–d)** and arterial phase postmortem CT angiography images in a 59-year-old woman who died of internal exsanguination shortly after Whipple surgery. **(a)** A large left-sided hemothorax with mediastinal shift to the right. During the surgery, supraceliac clamping of the abdominal aorta was performed to stop intraperitoneal bleeding. The clamp was later loosened but left in place (arrow in **b**). The fatal hemothorax was caused by hemorrhage from the left 11th intercostal artery, which was torn near its origin from the aorta just above the diaphragm during placement of the clamp. The contrast media extravasation from the artery’s origin, reaching cranially into the thorax (arrows in **b**, **c**, and **d**), is displayed on arterial phase postmortem CT angiographic images (**c** and **d**). This finding may have been difficult to detect at autopsy because of the small size and location of the vessel. In this case it could not be displayed at autopsy because of large amounts of intraperitoneal and intrathoracic clotted blood, and multiple previous abdominal operations with extensive scar tissue formation and adhesions.

**Figure 3d:**
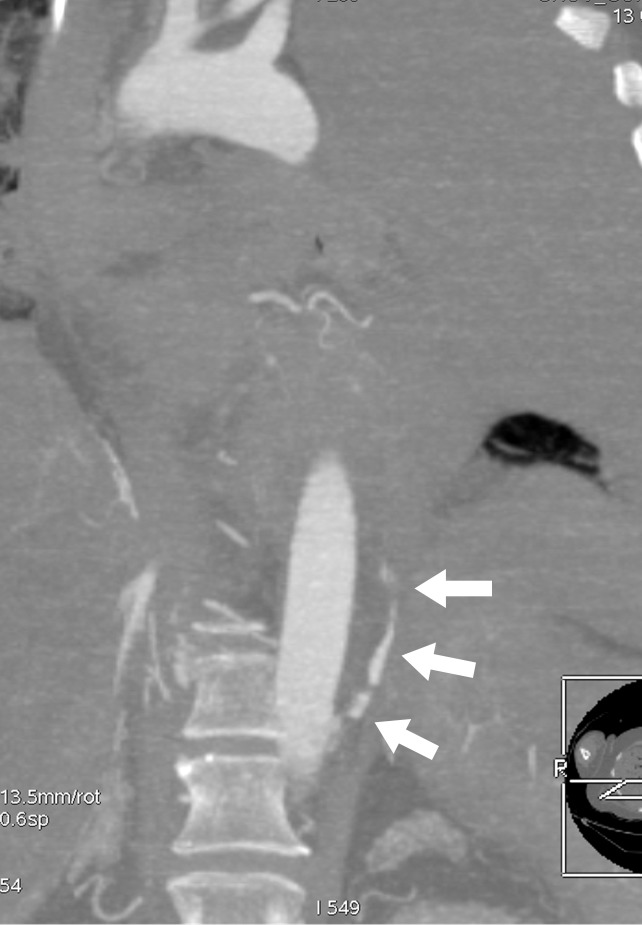
**(a)** Postmortem CT and **(b–d)** and arterial phase postmortem CT angiography images in a 59-year-old woman who died of internal exsanguination shortly after Whipple surgery. **(a)** A large left-sided hemothorax with mediastinal shift to the right. During the surgery, supraceliac clamping of the abdominal aorta was performed to stop intraperitoneal bleeding. The clamp was later loosened but left in place (arrow in **b**). The fatal hemothorax was caused by hemorrhage from the left 11th intercostal artery, which was torn near its origin from the aorta just above the diaphragm during placement of the clamp. The contrast media extravasation from the artery’s origin, reaching cranially into the thorax (arrows in **b**, **c**, and **d**), is displayed on arterial phase postmortem CT angiographic images (**c** and **d**). This finding may have been difficult to detect at autopsy because of the small size and location of the vessel. In this case it could not be displayed at autopsy because of large amounts of intraperitoneal and intrathoracic clotted blood, and multiple previous abdominal operations with extensive scar tissue formation and adhesions.

Our results also confirm the superior detection of bone lesions with postmortem CT compared with autopsy, whereas its detection rate for essential parenchyma, soft-tissue, and vascular lesions was significantly lower. The greatest number of findings in cases of natural death and suspected medical errors were missed at postmortem CT, in which half or more of the essential findings were missed. In both of these groups, the majority of pathologic findings were categorized as vascular or parenchymal, in which unenhanced CT is less sensitive. Interestingly, the overall detection rate of soft-tissue lesions was slightly higher at postmortem CT than it was at autopsy. However, this superiority is on the basis of useful and unimportant findings, whereas for essential soft-tissue findings autopsy was superior to postmortem CT. This result may be explained by the sensitivity of postmortem CT to pathologic changes in the subcutaneous fat. These lesions are less frequently detected or described at autopsy and only rarely considered essential.

Although these results should be interpreted with caution, they show that a number of essential findings are not detected at autopsy, especially bone and vascular lesions. This confirms the results of previous studies (14), and is also in line with our professional experience. In some cases, the interpretation of the autopsy results regarding the cause of death and events leading to death would have been incomplete or simply wrong if postmortem CT and postmortem CT angiography had not been performed.

We do not know if any potential findings were missed despite the use of both postmortem CT angiography and autopsy, and if they were missed, we do not know how many. However, even though we were unable to test postmortem CT angiography and autopsy against an independent reference standard (because none exists), our results show that the combination of postmortem CT angiography and autopsy is clearly superior to autopsy alone.

There has been concern in many countries about declining autopsy rates over recent decades. A high-quality postmortem examination is important not only in forensic cases, but also for the evaluation of the quality of clinical diagnosis and therapy in clinical pathologic analysis. It is thus an important instrument for both justice and medical quality control. The importance of both aspects cannot be overestimated. Postmortem CT and postmortem CT angiography might be feasible ways to increase the number of high quality postmortem examinations.

An efficient postmortem examination should be performed in a stepwise manner, beginning with a thorough external examination of the body and the circumstances of death. The next step would be to perform postmortem CT, which may be sufficient to confirm a suspected cause and manner of death. Even if the results are inconclusive, it acts as a triage tool because findings from postmortem CT help to determine whether autopsy, postmortem CT angiography, targeted histologic analysis, or any combination would be best to help ascertain the cause and manner of death. If used in this way, postmortem CT would in many cases shorten the length of postmortem examinations and reduce the effort required to perform them. The more time-consuming and more expensive techniques of postmortem CT angiography, autopsy, and histologic analysis would be reserved for ambiguous postmortem CT results. The number of high-quality postmortem examinations could be increased relatively easily to counter declining autopsy rates and the overall quality of postmortem examinations would increase.

Our study has limitations. In some institutes that participated in this study, postmortem CT and postmortem CT angiography were routine practice as a means of informing the autopsy. Therefore, in these examinations it was not possible to blind the forensic pathologists who performed the autopsy to the imaging results for both legal and ethical reasons. As a result, we expect that some findings were reported at autopsy that might otherwise have remained undetected, improving the apparent diagnostic ability of autopsy. Because we were unable to use a double-blind study design, we could not assess the accuracy of the final diagnosis of the cause of death. For the same reason, the sensitivity and specificity of the applied methods were not calculated because the value would not be sufficiently objective. Therefore, only the lesion detection rate was used for comparisons. This can be interpreted as sensitivity if the combination of autopsy with postmortem CT or postmortem CT angiography is considered the ground truth.

The study design enabled us to evaluate organ-specific findings, which can be identified by using different techniques. It did not focus on the advantages and limitations for specific case groups. Further studies focused on particular demands (eg, cardiovascular deaths, deaths in childhood, and deaths related to gunshots) and anatomic regions (eg, cardiac and cerebral) are required.

Our study leads to several important conclusions. A number of important findings remain unreported when postmortem CT, postmortem CT angiography, or autopsy are not considered in conjunction with each other. Indeed, postmortem CT angiography detects a greater number of important findings than autopsy, especially vascular and bone findings; it is therefore the method of choice for vascular findings. For parenchyma and soft-tissue findings, small differences are shown at autopsy and postmortem CT angiography, but postmortem CT alone is inferior. By combining autopsy and CT angiography, the reported number of findings can be increased, leading to a better postmortem examination. If only imaging or autopsy can be applied, the choice depends on the investigated case and the suspected findings.

**Summary**

If autopsy had been performed without postmortem CT, 39% of all findings and 23% of essential findings would not have been reported.

**Implications for Patient Care**

■ Postmortem CT angiography is superior to autopsy and CT without angiography to help detect forensically essential findings.
■ The combination of autopsy and multiphase CT angiography helps to reveals most findings.

## SUPPLEMENTAL TABLES

Tables E1–E3 (PDF)
