# Supplementary material for: Postmortem CT Angiography Compared with Autopsy: A Forensic Multicenter Study
Source: Radiology. 2018 May 1;288(1):270–6. doi: 10.1148/radiol.2018170559 (PMC6027995; doi:10.1148/radiol.2018170559)
Supplement: Tables E1–E3 (PDF) [file ry170559suppa1.pdf]

©RSNA, 2018
10.1148/radiol.2018170559

Table E2: Number and Distribution of Cases by Site

	Center 1 (*n* = 108)	Center 2 (*n* = 81)	Center 3 (*n* = 111)	Center 4 (*n* = 15)	Center 5 (*n* = 26)	Center 6 (*n* = 51)	Center 7 (*n* = 59)	Center 8 (*n* = 28)	Center 9 (*n* = 21)
Natural death	51	18	72	7	2	36	38	17	17
Polytrauma	18	9	2	0	9	2	5	1	0
Other violent death	28	53	4	8	15	12	15	4	3
Suspected medical error	11	1	33	0	0	1	1	6	1

Table E3a: Stratification of Results across Sites for Natural Death

Natural death					
		Essential (*n* = 1348)	Useful (*n* = 4080)	Unimportant (*n* = 2447)	Total
Bone (*n* = 2239)					
	PMCT	85% (11/13)	90% (1410/1560)	99% (657/666)	93% (2078/2239)
	MPMCTA	85% (11/13)	91% (1417/1560)	99% (658/666)	93% (2086/2239)
	Autopsy	54% (7/13)	46% (711/1560)	15% (103/666)	37% (821/2239)
Parenchyma (*n* = 2999)					
	PMCT	58% (398/690)	71% (777/1087)	76% (933/1222)	70% (2108/2999)
	MPMCTA	81% (559/690)	86% (935/1087)	81% (993/1222)	83% (2487/2999)
	Autopsy	86% (593/690)	69% (753/1087)	59% (721/1222)	69% (2067/2999)
Soft Tissue (*n* = 575)					
	PMCT	90% (45/50)	86% (215/251)	66% (182/274)	77% (442/575)
	MPMCTA	90% (45/50)	92% (232/251)	80% (219/274)	86% (496/575)
	Autopsy	64% (32/50)	63% (157/251)	38% (104/274)	51% (293/575)
Vascular (*n* = 2062)					
	PMCT	22% (131/595)	67% (793/1182)	64% (181/285)	54% (1105/2062)
	MPMCTA	91% (540/595)	96% (1136/1182)	93% (266/285)	94% (1942/2062)
	Autopsy	79% (472/595)	75% (882/1182)	63% (180/285)	74% (1534/2062)
Total (*n* = 7875)					
	PMCT	43% (585/1348)	78% (3195/4080)	80% (1953/2447)	73% (5733/7875)
	MPMCTA	86% (1155/1348)	91% (3720/4080)	87% (2136/2447)	89% (7011/7875)
	Autopsy	82% (1104/1348)	61% (2503/4080)	45% (1108/2447)	60% (4715/7875)

Table E3b: Stratification of Results across Sites for Violent Death

Violent death					
		Essential (*n* = 1742)	Useful (*n* = 1704)	Unimportant (*n* = 1217)	Total
Bone (*n* = 1439)					
	PMCT	91% (258/283)	91% (762/837)	97% (310/319)	92% (1330/1439)
	MPMCTA	94% (265/283)	92% (773/837)	97% (310/319)	94% (1348/1439)
	Autopsy	65% (184/283)	67% (563/837)	5% (16/319)	53% (763/1439)
Parenchyma (*n* = 1493)					
	PMCT	76% (451/591)	70% (306/435)	68% (316/467)	72% (1073/1493)
	MPMCTA	91% (535/591)	84% (366/435)	77% (358/467)	84% (1259/1493)
	Autopsy	81% (478/591)	66% (286/435)	63% (295/467)	71% (1059/1493)
Soft Tissue (*n* = 1085)					
	PMCT	69% (491/712)	70% (187/267)	50% (53/106)	67% (731/1085)
	MPMCTA	89% (631/712)	86% (230/267)	68% (72/106)	86% (933/1085)

	Autopsy	90% (642/712)	60% (159/267)	49% (52/106)	79% (853/1085)
Vascular (*n* = 646)					
	PMCT	12% (19/156)	58% (96/165)	74% (240/325)	55% (355/646)
	MPMCTA	99% (155/156)	99% (164/165)	95% (308/325)	97% (627/646)
	Autopsy	39% (61/156)	62% (102/165)	73% (238/325)	62% (401/646)
Total (*n* = 4663)					
	PMCT	70% (1219/1742)	79% (1351/1704)	76% (919/1217)	75% (3489/4663)
	MPMCTA	91% (1586/1742)	90% (1533/1704)	86% (1048/1217)	89% (4167/4663)
	Autopsy	78% (1365/1742)	65% (1110/1704)	49% (601/1217)	66% (3076/4663)

Table E3c: Stratification of Results across Sites for Polytrauma

Polytrauma					
		Essential (*n* = 977)	Useful (*n* = 2509)	Unimportant (*n* = 360)	Total
Bone (*n* = 2331)					
	PMCT	97% (341/350)	97% (1869/1935)	96% (44/46)	97% (2254/2331)
	MPMCTA	99% (345/350)	97% (1871/1935)	96% (44/46)	97% (2260/2331)
	Autopsy	66% (231/350)	52% (1009/1935)	22% (10/46)	54% (1250/2331)
Parenchyma (*n* = 811)					
	PMCT	85% (338/400)	65% (154/237)	60% (105/174)	74% (597/811)
	MPMCTA	95% (379/400)	81% (191/237)	65% (113/174)	84% (683/811)
	Autopsy	74% (297/400)	71% (169/237)	74% (129/174)	73% (595/811)
Soft Tissue (*n* = 432)					
	PMCT	85% (93/109)	76% (201/264)	44% (26/59)	74% (320/432)
	MPMCTA	93% (101/109)	86% (226/264)	44% (26/59)	82% (353/432)
	Autopsy	77% (84/109)	62% (164/264)	71% (42/59)	67% (290/432)
Vascular (*n* = 272)					
	PMCT	6% (7/118)	25% (18/73)	79% (64/81)	33% (89/272)
	MPMCTA	100% (118/118)	86% (63/73)	91% (74/81)	94% (255/272)
	Autopsy	31% (37/118)	30% (22/73)	80% (65/81)	46% (124/272)
Total (*n* = 3846)					
	PMCT	80% (779/977)	89% (2242/2509)	66% (239/360)	85% (3260/3846)
	MPMCTA	97% (943/977)	94% (2351/2509)	71% (257/360)	92% (3551/3846)
	Autopsy	66% (649/977)	54% (1364/2509)	68% (246/360)	59% (2259/3846)

Table E3d: Stratification of Results across Sites for Medical Error

Medical error					
		Essential (*n* = 326)	Useful (*n* = 1247)	Unimportant (*n* = 698)	Total
Bone (*n* = 564)					
	PMCT	75% (3/4)	91% (341/374)	100% (186/186)	94% (530/564)
	MPMCTA	100% (4/4)	91% (341/374)	100% (186/186)	94% (531/564)
	Autopsy	75% (3/4)	53% (200/374)	22% (41/186)	43% (244/564)
Parenchyma (*n* = 761)					
	PMCT	53% (74/139)	78% (254/325)	79% (234/297)	74% (562/761)
	MPMCTA	*76%* (105/139)	87% (282/325)	83% (246/297)	83% (633/761)
	Autopsy	82% (114/139)	73% (238/325)	53% (157/297)	67% (509/761)
Soft Tissue (*n* = 279)					
	PMCT	92% (45/49)	91% (133/146)	61% (51/84)	82% (229/279)
	MPMCTA	96% (47/49)	92% (135/146)	77% (65/84)	88% (247/279)
	Autopsy	90% (44/49)	74% (108/146)	45% (38/84)	68% (190/279)
Vascular (*n* = 667)					
	PMCT	30% (41/134)	64% (258/402)	59% (77/131)	56% (376/667)

	MPMCTA	93% (125/134)	97% (388/402)	97% (127/131)	96% (640/667)
	Autopsy	63% (85/134)	70% (282/402)	56% (73/131)	66% (440/667)
Total ($n = 2271$)					
	PMCT	50% (163/326)	79% (986/1247)	79% (548/698)	74% (1697/2271)
	MPMCTA	86% (281/326)	92% (1146/1247)	89% (624/698)	90% (2051/2271)
	Autopsy	75% (246/326)	66% (828/1247)	44% (309/698)	61% (1383/2271)

Table E1: Acquisition Parameters for Postmortem CT Angiography

		Scan type	Slice thickness Table speed Pitch	Spacing interval (mm)	Scan Field Of View (FOV)	kilo Volts (kV)	milli Amperage mA (auto-mA)	Algorithm of reconstruction
Center 1 GE-Health Care Light Speed-8 rows	Arterial phase	Helical 0.8s	1.25 mm 13.50 1.35:1	0.6	500 mm	120	100–280	standard
	Venous phase	Helical 0.8s	1.25 mm 13.50 1.35:1	1	500 mm	120	100–280	standard
	Dynamic phase	Helical 0.8s	2.5 mm 13.50 1.35:1	2	500 mm	120	100–280	standard
Center 2 GE-Bright Speed-16 rows	Arterial phase	Helical 0.1s	1.25 mm 9.37 0.938:1	1	500 mm	120	190–380	standard
	Venous phase	Helical 0.1s	1.25 mm 9.37 0.938:1	1	500 mm	120	190–380	standard
	Dynamic phase	Helical 0.1s	1.25 mm 9.37 0.938:1	1	500 mm	120	190–380	standard
Center 3 GE-Health Care Light Speed Discovery CT750 HD	Arterial phase	Helical 0.5s	0.75 mm 19.2 mm/s 0.80:1	0.7	414 mm	120	157	standard
	Venous phase	Helical 0.5s	1.5 mm 19.2 mm/s 0.80:1	1	414 mm	120	157	standard
	Dynamic phase	Helical 0.5s	0.75 mm 19.2 mm/s 0.80:1	2	414 mm	120	157	standard
Center 4 Philips Brilliance-16 rows	Arterial phase	Helical 16*0,75	1.0 mm 15 0,938	0.8/1*	500 mm	120	200	standard
	Venous phase	Helical 16*0,75	1.0 mm 15 0,938	1	500 mm	120	200	standard
	Dynamic phase	Helical 16*0,75	1.0 mm 15 0,938	0.8/1	500 mm	120	200	standard
Center 5 Siemens Somatom Emotion-16 rows	Arterial phase	Helical 1s	1.5 mm 19.2 0.9	1	500 mm	110	(250)	standard
	Venous phase	Helical 1s	2.0 mm 19.2 0.7	1.5	500 mm	110	(200)	standard
	Dynamic phase	Helical 1s	2.0 mm 19.2 0.7	1.5	500 mm	110	(200)	standard
Center 6 Toshiba Aquilion 64 × 0.5 mm slice scanner	Arterial phase	Helical 0.5s rotation	1 mm 26.5 mm 0.83:1	0.8 mm	500 mm	120	300mA	standard
	Venous phase	Helical 0.5s rotation	1 mm 26.5 mm 0.83:1	0.8 mm	500 mm	120	300mA	standard
	Dynamic phase	Helical 0.5s rotation	1 mm 26.5 mm 0.83:1	0.8 mm	500 mm	120	300mA	standard
Center 7 Siemens Somatom Emotion 16 scanner	Arterial phase	Helical 0.6s rotation	1.5 mm 12.5 0.8:1	1.5 mm	500 mm	130	Auto CareDose4D	standard
	Venous phase	Helical 0.6s rotation	1.5 mm 12.5 0.8:1	1.5 mm	500 mm	130	Auto CareDose4D	standard
	Dynamic phase	Helical 0.6s rotation	1.5 mm 12.5 0.8:1	1.5 mm	500 mm	130	Auto CareDose4D	standard
Center 8 Siemens Sensation-16 rows	Arterial phase	Helical 0.5s	0.75 mm 19.2 mm/S 0.80:1	0.7	414 mm	120	157	standard
	Venous phase	Helical 0.5s	1.5 mm 19.2 mm/S 0.80:1	1	414 mm	120	157	standard
	Dynamic phase	Helical 0.5s	0.75 mm 19.2 mm/S 0.80:1	2	414 mm	120	157	standard
Center 9 Philips Brilliance-2 rows	Arterial phase	axial (head), helical (neck, thorax, abdomen)	2 mm (head), 3 mm (thorax + abdomen)	1.5	500 mm	120	150–250	standard
	Venous phase	axial (head), helical (neck, thorax, abdomen)	2 mm (head), 3 mm (thorax + abdomen)	1.5	500 mm	120	150–250	standard
	Dynamic phase	axial (head), helical (neck, thorax, abdomen)	2 mm (head), 3 mm (thorax + abdomen)	1.5	500 mm	120	150–250	standard

* 0.8 mm for CCT and thorax; 1 mm for the remaining parts of the body.
